# Microbial influence on the larval survival of Japanese eel *Anguilla japonica*: Antibiotic-mediated alterations and biomarker isolation

**DOI:** 10.1371/journal.pone.0306634

**Published:** 2024-07-08

**Authors:** Youhei Fukui, Yoji Nakamura, Hitoshi Imaizumi, Masaaki Kamoshida

**Affiliations:** 1 Fisheries Technology Institute, Japan Fisheries Research and Education Agency, Minamiise, Japan; 2 Fisheries Resources Institute, Japan Fisheries Research and Education Agency, Yokohama, Japan; 3 Fisheries Technology Institute, Japan Fisheries Research and Education Agency, Minamiizu, Japan; 4 Headquarters, Japan Fisheries Research and Education Agency, Yokohama, Japan; Tanta University Faculty of Agriculture, EGYPT

## Abstract

In rearing systems for the Japanese eel *Anguilla japonica*, although it is assumed that microorganisms influence larval survival and mortality, particularly during the early stages of growth, the effects of bacterial communities on larval survival have yet to be sufficiently determined. In this study, we compared the bacterial communities associated with larval survival at three stages of eel growth. To artificially alter bacterial communities and assess larval survival, eel larvae were treated with 11 types of antibiotic, and larval survival and bacterial characteristics were compared between the antibiotic-treated and antibiotic-free control groups. Throughout the three growth stages, eels treated with four antibiotics (polymyxin B, tetracycline, novobiocin, and erythromycin) had survival rates higher than those in the control groups. The bacterial communities of surviving larvae in the control and antibiotic groups and dead larvae in the control groups were subsequently analyzed using 16S rRNA gene amplicon sequencing. PERMANOVA analysis indicated that these three larval groups were characterized by significantly different bacterial communities. We identified 14 biomarker amplicon sequence variants (ASVs) of bacterial genera such as *Oceanobacter*, *Alcanivorax*, *Marinobacter*, *Roseibium*, and *Sneathiella* that were enriched in surviving larvae in the antibiotic treatment groups. In contrast, all four biomarker ASVs enriched in dead larvae of the control groups were from bacteria in the genus *Vibrio*. Moreover, 52 bacterial strains corresponding to nine biomarkers were isolated using a culture method. To the best of our knowledge, this is the first study to evaluate the bacterial communities associated with the survival and mortality of larvae in during the early stages of Japanese eel growth and to isolate biomarker bacterial strains. These findings will provide valuable insights for enhancing larval survival in the eel larval rearing systems from a microbiological perspective.

## Introduction

Marine fish rearing systems facilitate a strong association between the microbiota and larvae (i.e., across the egg and larval stages). This association ultimately affects the establishment of normal microflora or culminates in disease outbreaks [1, 2]. Moreover, in larval rearing, detrimental larvae-microbe interactions result in significant losses in larval viability and quality [3, 4]. Therefore, controlling microflora for efficient larval development could inhibit the proliferation of opportunistic pathogenic bacteria, enhancing larval growth and survival [5]. Efficient microbial management strategies are also required to minimize problems encountered during intensive production or larval rearing [2, 4, 5]. However, developing such strategies requires a comprehensive evaluation of the physiological effects of various microbial communities on the host [3]. Such an approach will allow for the development of more targeted solutions that do not negatively impact larval production in the long run.

The Japanese eel, *Anguilla japonica*, is one of the most important aquaculture species in East Asia. Almost all *Anguilla* seeds used in eel farming worldwide are dependent on wild-caught glass eels [6]. Owing to a decline in natural eel stocks and an increase in the price of glass eels, artificial rearing of leptocephali and grass eels has been conducted to ensure that the full life cycle is achieved in captivity [7–9]. Currently, further development of larval rearing technologies will be necessary for the sustainable mass production of artificial eel seedlings [10]. For the healthy growth of the eel larvae until metamorphosis, the maintenance of water quality, in addition to the cleanliness of tank walls, has been shown to be of utmost importance [10]. Specifically, the adhesion of food on tank walls has been shown to easily drive bacterial contamination, directly impacting the survival of eel larvae [11]. To address these microbiological problems, larval rearing systems employ techniques that entail flushing residual food out from the tanks and the daily replacement of used tanks [8, 10].

Microbiological studies on the Japanese eel have primarily focused on pathogens in farmed adults, culminating in the identification of several bacteria as pathogens, including *Vibrio vulnificus* [12], *Aeromonas salmonicida* [13, 14], *Edwardsiella tarda* [15], *Nocardia seriolae* [16], and *Pseudomonas anguilliseptica* [17]. Contrastingly, however, there have been only two studies that have assessed the effects of harmful or pathogenic bacteria on the larvae of Japanese eels. In these, bacterial isolates closely related to the genera *Lacinutrix*, *Crocinitomix*, and *Pseudoalteromonas* were established to cause mortality in eel larvae at 10 days post-hatching (dph) [18]. Recently, *Aureispira anguillae* was identified as a novel pathogenic bacterium associated with eel leptocephali [19]. In the European eel *Anguilla anguilla*, harmful bacterial communities and larval immune status has been shown to be associated with a sharp decrease in early survival [20]. These studies did not clearly compare the bacterial isolates and communities between surviving and dead eel larvae; hence, it is unknown whether bacteria are beneficial or harmless to the survival of eel larvae. Regarding the use of antimicrobial agents, the addition of silver nitrate to seawater used to rear Japanese eels significantly increased larval survival rates [21]. In addition, in the context of the European eel, whose egg and larval production are negatively affected by microbial interference, the use of antibiotics and disinfectants has been shown to significantly improve larval hatching and subsequent survival rates [22]. These observations led us to speculate that antimicrobial agents may reduce the abundance of bacterial communities that negatively affect survival while increasing the abundance of those that positively impact survival. The use of various antibiotics to isolate the bacteria responsible for the high survival rate of eel larvae would be effective in discovering diverse and unique bacterial communities that are usually hidden in eel larvae. In addition, in laboratory rearing using well plates, the bacteria present near eel larvae are expected to have a direct effect on their survival.

In this study, we aimed to investigate the survival of Japanese eel larvae after receiving antibiotic treatments in the laboratory. We compared the bacterial characteristics of individuals that exhibited survival and mortality phenotypes. We evaluated the bacterial communities, abundance, and isolation using 16S rRNA gene amplicon sequencing and culture methods. Additionally, we investigated the effects of bacterial communities on the survival of eel larvae at different growth stages (5, 20, and 40 dph).

## Materials and methods

### Eel larval samples

Hatched larvae were reared without being fed until 4 dph at Shibushi Station following the previously established protocols [23]. Larvae at Minamiizu Station were reared until 18 and 38 dph, according to previous protocols [7]; these larvae were fed a slurry-type artificial diet [24]. One day before the experiment, all the larvae were transported from the two stations to our laboratory. The larvae were transported in seawater containing 50 and 35 mg/L streptomycin and penicillin G at concentrations, respectively. The inclusion of these antibiotics was intended to prevent larval mortality during transport. Supplying seawater was also transported by a cool express from the Minamiizu Station and used to rear eel larvae. These larvae (5, 19, and 39 dph) were received the next day and used in subsequent survival experiments. All fish used in the present study were handled in accordance with the guidelines for animal experimentation of the Japan Fisheries Research and Education Agency.

### Survival experiments of eel larvae

The larvae were washed thrice using sterile artificial seawater (marine art SF-1; Tomita Pharmaceutical, Tokushima, Japan). Briefly, 9.8 mL of the supplying seawater was added to each well of a 6-well plate (BM Equipment, Tokyo, Japan). To circumvent bacterial contamination from dead larvae, only one larva was placed per well in the laboratory. This also allowed a comparison of the bacteria-related dynamics of surviving and dead larvae. Each larva, along with 200 μL of seawater, was transferred individually to each well using pipette tips. Eel larvae at 19 and 39 dph were acclimated in supplying seawater at 23°C 24 h prior to antibiotic treatment. The larvae at 5 dph were used in the survival experiments without acclimation. The experimental groups consisted of 11 antibiotics (Nacalai Tesque, Kyoto, Japan) and two control solvents, distilled water and ethanol. The antibiotics used included ampicillin (AP), penicillin G (PC), polymyxin B (PM), streptomycin (SM), kanamycin (KM), neomycin (NM), tetracycline (TC), novobiocin (NB), nalidixic acid (NA), chloramphenicol (CP), and erythromycin (EM). The first nine antibiotics were dissolved in distilled water, and the last two were dissolved in ethanol. These antibiotic solutions were filtered through a membrane filter with a pore size of 0.2 μm (Advantec, Tokyo, Japan). Ten larvae were used for each experimental group. The antibiotic solutions were added to their respective wells at a concentration of 40 mg/L; distilled water (DW) and ethanol (ET) without antibiotics were used as controls. The experiments were conducted at 23°C. The larvae were not fed, and the seawater was not replaced throughout the experiment. The experiment was terminated when half of the larval population (more than five individuals) in the control groups died to match the survival conditions of the larvae used in three independent experiments. The experiments were repeated three times using eel larvae at 5, 20, and 40 dph, hatched from different eel parents. The average rearing periods for the 5, 20, and 40 dph larvae were 1, 2.7, and 3.7 days, respectively. The statistical differences between the control and antibiotic groups were assessed using Dunnett’s test (*p* < 0.05).

### Sample collection

Regarding the 16S rRNA gene metagenomic analysis and cultivation, surviving larvae were collected in both antibiotic and control groups, and dead larvae were exclusively from the control groups. Specifically, larval samples were collected from the following three major groups: i) surviving larvae in the four antibiotic groups (PM, TC, NB, and EM) with significantly higher survival rates than those in the control groups, ii) surviving larvae in the control groups (DW and ET), and iii) dead larvae in the control groups (DWD and ETD). These samples were obtained from larvae at three different growth stages. During the rearing period, larval survival in each well was observed daily, and when larvae in the control groups died, dead individuals and the surrounding seawater were collected (DWD and ETD). At the end of the experiments, surviving larvae and the surrounding seawater were collected from the control (DW and ET) and four antibiotic groups (PM, TC, NB, and EM). Larvae from the same experimental group were pooled, and seawater associated with the larval collection was removed and replaced with sterile seawater (0.5 or 1 mL/larva). The samples were thoroughly mixed using a Vortex-Genie 2 mixer (Scientific Industries, Bohemia, NY, USA) for 5 min, and the suspension was divided into two subsamples that were used for bacterial isolation and DNA extraction for amplicon sequencing analysis.

### 16S rRNA gene amplicon sequencing analysis

The larval sample suspension was first filtered through a 0.2 μm pore size Nuclepore filter (Merck, Darmstadt, Germany). Genomic DNA was isolated from the filters using the DNeasy PowerSoil Kit (Qiagen, Venlo, Netherlands). The filtered samples were directly immersed in PowerBead tubes and extracted according to the manufacturer’s instructions. The concentration of extracted DNA was measured using a Qubit 3.0 Fluorometer (Thermo Fisher Scientific, Waltham, MA, USA). The first PCR was performed using a primer set for V3V4f (341f) and V3V4r (805r) to target the V3–V4 region of the 16S rRNA gene. The PCR reaction was performed at 94°C for 2 min, followed by 25 cycles of 95°C for 30 s, 55°C for 30 s, 72°C for 30 s, and a final extension of 72°C for 5 min, and electrophoresis was conducted to verify PCR products. The subsequent PCR, library preparation, and 16S rRNA gene sequencing were outsourced to the Bioengineering Lab. Co., Ltd. (Kanagawa, Japan). Libraries were paired-end sequenced at 2 × 300 bp using a MiSeq sequencer (Illumina, San Diego, CA, USA).

### Data analysis

General data analysis of the amplicon sequences was performed using QIIME2. After removing chimeric and noisy sequences, amplicon sequence variance (ASV) and representative sequences of each ASV were determined using DADA2. The total number of reads obtained from 68 samples was 1,812,676 (average: 26,657), comprising 861 ASVs (S1 Table). The taxonomic identities of the ASVs were assigned using the SILVA database (version 138). Alpha diversity of the observed ASVs and Shannon indices were calculated at a minimum depth of 16,400 sequence reads. Alpha diversity data were analyzed using ANOVA, followed by Tukey’s test. Principal coordinate analysis (PCoA) of the bacterial community structures was performed using the Bray–Curtis distance and visualized using Emperor and Qiime2 View. Permutational multivariate analysis of variance (PERMANOVA) was performed to test the Bray–Curtis dissimilarity among the sample groups, which were grouped by larval status or growth stage, using the R package *vegan* with 999 permutations. For a group setting in which at least one pair of groups was significantly dissimilar, a pairwise PERMANOVA with Bonferroni’s correction was conducted using the R package *pairwiseAdonis* (*p* < 0.05). A heatmap of the top 30 most abundant ASVs from all larval samples was generated using the R package *heatmap3*. The relative abundance at the ASV level was represented by the z-score and the dendrogram was calculated based on the complete linkage method. To extract the biomarker ASVs, the number of reads obtained from surviving larvae in the antibiotic and control groups was compared to those obtained from dead larvae in the control groups by Dunnett’s test (*p* < 0.05) using the R package *SimComp*. Among ASVs with significant differences, those with > 500 reads were used as biomarkers.

### Cultivation and enumeration of marine bacteria

Bacteria obtained from dead larvae and seawater samples in the control groups were cultivated throughout the experimental period, and those obtained from surviving larvae and seawater samples in the control and antibiotic groups were cultured at the end of the experiments. A subsample of the suspension obtained previously (see ‘Sample collection’ subsection) was diluted 10-fold using sterilized seawater, and each dilution was plated on Marine Agar 2216 (MA; Becton, Dickinson and Company, Franklin Lakes, NJ, USA). The plates were incubated at 20°C for 7 days. The number of marine bacterial colonies grown on MA was counted, and the viable counts of marine bacteria were determined. Detection limits of bacterial counts for samples free of colonies were then set as 5 CFU/mL or 2.5 CFU/eel. After the bacterial counts were converted to log_10_ values, the average counts from three independent experiments were used for statistical analysis. The numbers of marine bacteria in the surviving larvae and seawater of the control and antibiotic groups were compared with those of dead larvae and seawater in the control groups using Dunnett’s test or t-test (*p* < 0.05).

### Sequencing of 16S rRNA gene and *pyrH* gene of bacterial isolates

Dominant bacteria were isolated from samples of both surviving (DW and ET) and dead larvae (DWD and ETD) in the control groups and from surviving larvae in the four antibiotic groups (PM, TC, NB, and EM). If no colonies appeared on MA from larval samples, dominant bacteria were isolated from seawater samples. Based on colony morphology and color, one or two representative colonies were selected from the dominant colonies in each experimental group. To identify the bacterial isolates, genomic DNA extraction and 16S rRNA gene sequencing were performed, as previously described [25]. Briefly, the genomic DNA from a single colony was extracted using Tris-EDTA buffer containing Triton-X100 and chloroform-isoamyl alcohol. The 16S rRNA gene was amplified using primers 27F and 1492R and then sequenced using the 27F primer to determine approximately 700 bp of the first half region of the 16S rRNA gene. The sequences of all isolates were grouped based on 100% similarity, and a representative strain was then selected from each group. The nearly full-length 16S rRNA gene of these representative strains was determined by further sequencing using primers 27F, 515F, 800F, 1190F, 530R, 798R, and 1492R. These sequences were subjected to NCBI BLASTN in the GenBank database and compared with sequences of known type strains. Species identification of isolates was based on > 99% similarity with the previously described type species [26]. To determine whether the sequences of the representative strains corresponded to ASVs obtained through amplicon analysis, we confirmed if both sequences shared 100% similarity using GENETYX version 15 (Genetyx Corporation, Tokyo, Japan).

The 16S rRNA gene sequences of *Vibrio* species are highly similar, making species-level discrimination difficult [27]. Therefore, isolates identified via 16S rRNA gene, as part of the genus *Vibrio* were sequenced for the *pyrH* (uridylate kinase) gene, which allows for a fine-scale and accurate identification of species [28, 29]. The gene was amplified and sequenced using primers pyrH80F and pyrH530R [27]. Species identification of *Vibrio* isolates was determined by more than 98.4% similarity to known type species of *Vibrio* [30].

## Results

### Survival rates of eel larvae in rearing experiments

**Fig 1:**
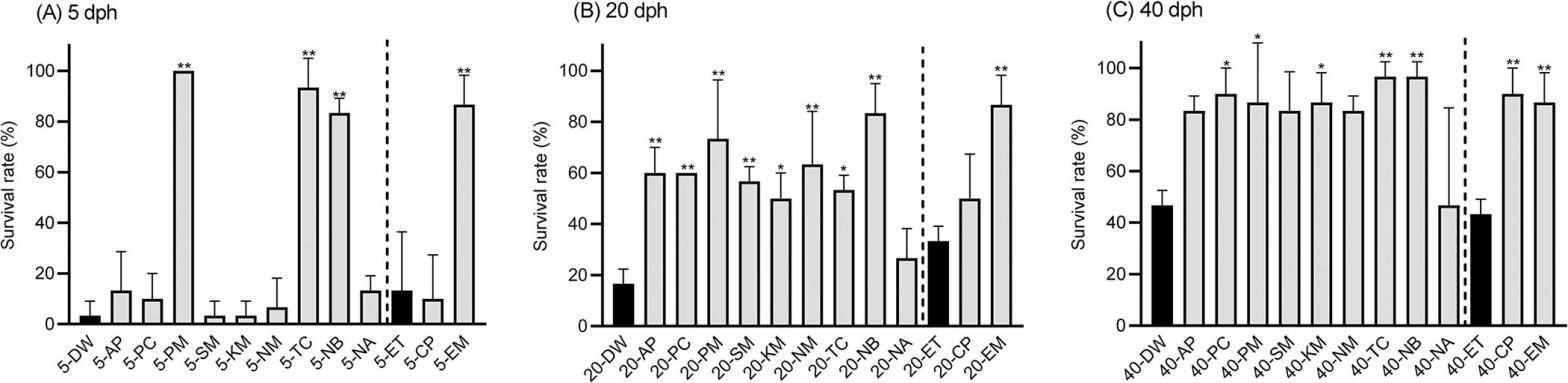
Survival rates of eel larvae at three growth stages. (A) 5 dph, (B) 20 dph, and (C) 40 dph. The survival rates of AP, PC, PM, SM, KM, NM, TC, NB, and NA were compared to those of DW, whereas the survival rates of CP and EM were compared to those of ET. Data represent the mean ± standard deviation (n = 3), and the asterisks on the bars show significant differences (* *p* < 0.05; ** *p* < 0.01).

For 5 dph larvae, larval survival rates in antibiotic groups of 5-PM, 5-TC, 5-NB, and 5-EM (100.0, 93.3, 83.3, and 86.7%; *p* < 0.01), except the other seven antibiotics, were significantly higher than those of the control groups (5-DW and 5-ET) (Fig 1A). In 20 dph larvae, the survival rates of nine antibiotic groups, except 20-NA and 20-CP, were significantly higher (50.0–86.7%; *p* < 0.05) than those of the control groups (20-DW and 20-ET) (Fig 1B). At 40 dph larvae, the survival rates of seven antibiotic groups (86.7–96.7%; *p* < 0.05), except 40-AP, 40-SM, 40-NM, and 40-NA, were significantly higher than those of the control groups (40-DW and 40-ET) (Fig 1C). Throughout the three growth stages, the four antibiotic groups (PM, TC, NB, and EM) had survival rates higher than those of the control groups.

### Bacterial diversity in eel larval samples

**Fig 2:**
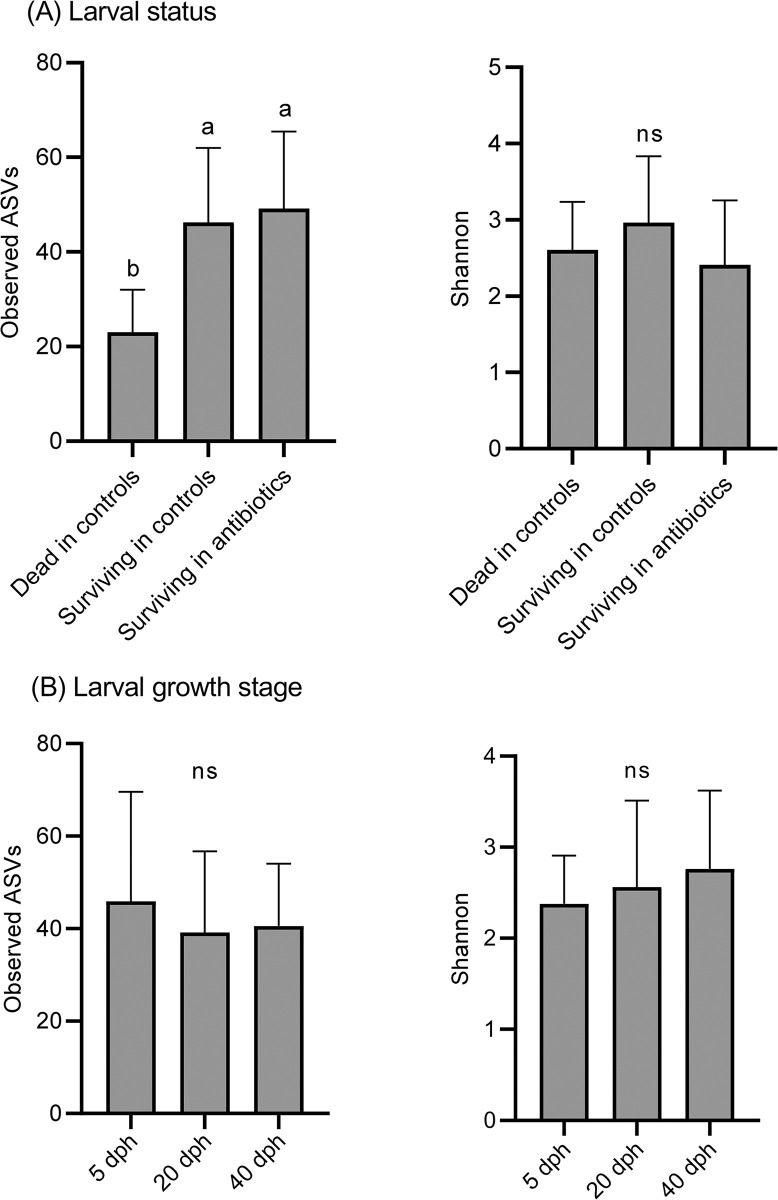
Alpha diversity indices of bacterial richness and evenness. (A) larval status and (B) growth stages. Data represent the mean ± standard deviation (n = 14–36/group), and the different letters on the bars show statistical differences (*p* < 0.01). ns, not significant.

**Fig 3:**
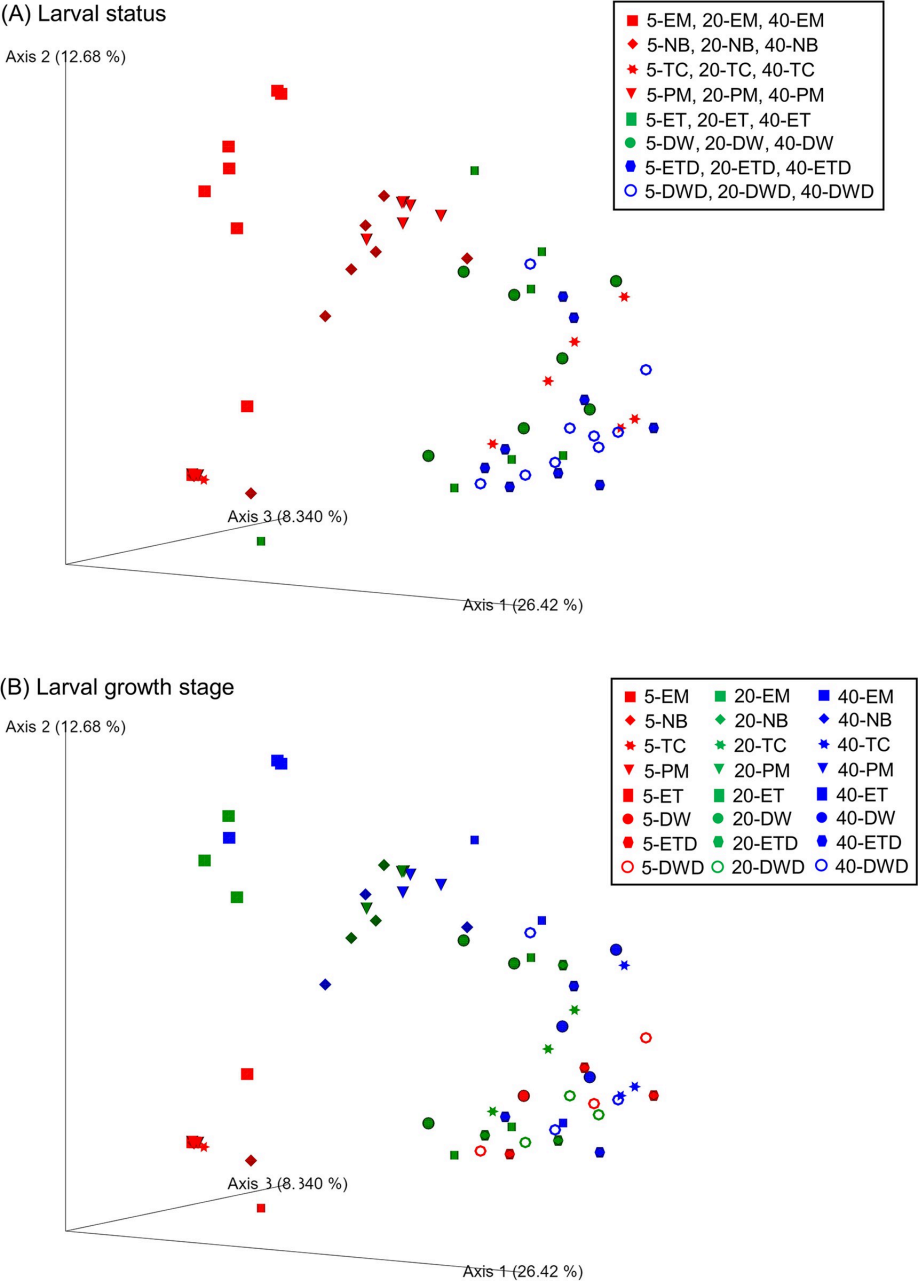
Principal coordinate analysis based on Bray–Curtis dissimilarities of bacterial communities hosted by eel larvae. (A) larval status and (B) growth stages. In (A), red, green, and blue denote surviving larvae in antibiotic groups, surviving larvae in control groups, and dead larvae in control groups, respectively. In (B), red, green, and blue denote 5, 20, and 40 dph of eel larvae, respectively.

Alpha diversity indices of richness and evenness were compared among the larval status and growth stages (Fig 2). Among larval status, the observed ASVs index of dead larvae in the control groups was significantly lower than that of surviving larvae in the control and antibiotic groups (*p* < 0.01) (Fig 2A). However, no significant differences in the alpha diversity indices were observed among the larval growth stages (Fig 2B). The differences in bacterial community structures among the larval status and growth stages were illustrated using PCoA (Fig 3). The plot based on the larval status showed that two main clusters of surviving larvae in the antibiotic groups and dead larvae in the control groups were formed and most surviving larvae in the control groups occurred between the two clusters (Fig 3A). PERMANOVA revealed a significant difference in the larval status (*p* = 0.001). Furthermore, pairwise PERMANOVA showed that all larval pairs, including “surviving in antibiotic groups vs. surviving in control groups,” “surviving in antibiotic groups vs. dead in control groups,” and “surviving in control groups vs. dead in control groups,” were also significantly dissimilar (*p* = 0.003–0.006). When comparing the different growth stages, some of the 5 dph plots tended to converge, whereas the 20 and 40 dph plots tended to diverge (Fig 3B). A significant difference was also observed in the growth stages (*p* = 0.001), and the pairwise PERMANOVA revealed that larval stage of 5 dph was significantly different from both 20 and 40 dph (*p* = 0.003), whereas the difference between 20 and 40 dph larvae was not significant (*p* = 0.054).

### Bacterial communities obtained from surviving and dead eel larvae

**Fig 4:**
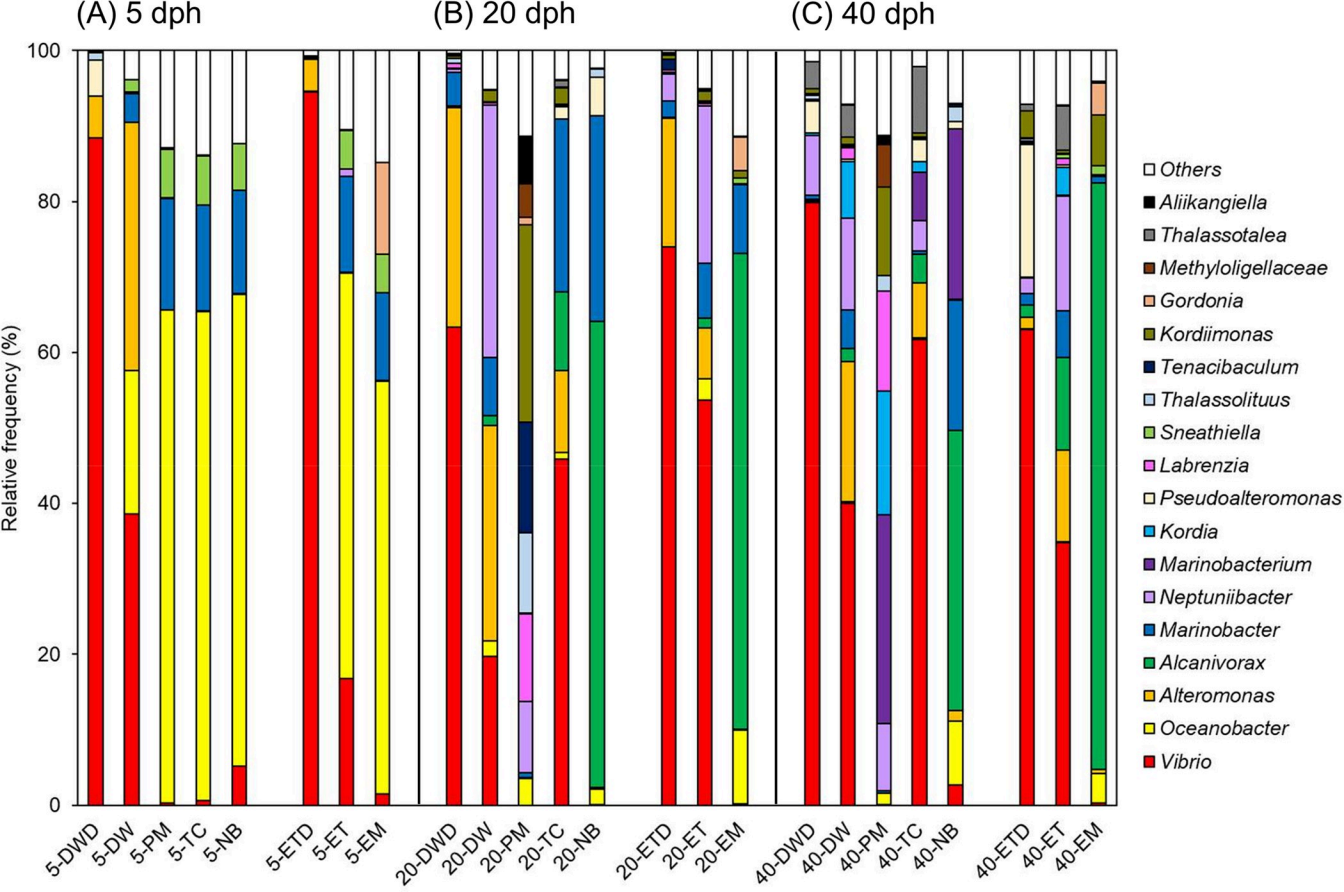
Bacterial community structure at family levels in surviving and dead eel larvae at three growth stages. (A) 5 dph, (B) 20 dph, and (C) 40 dph. The averages are presented from three independent trials. Genera that are present at average abundance > 5% are shown.

The bacterial communities obtained from surviving and dead larvae at 5, 20, and 40 dph were present at the genus level (Fig 4). In the control groups, the dominant genera (> 10% composition) of surviving larvae were *Vibrio* (16.8–38.6%) and *Oceanobacter* (19.0–53.7%) at 5 dph (5-DW and 5-ET) and *Vibrio* (19.7–53.7%) and *Neptuniibacter* (12.1–33.4%) at 20 and 40 dph (20-DW, 20-ET, 40-DW, and 40-ET). In contrast, most of the dead larvae (DWD and ETD) were associated with *Vibrio* (63.0–94.5%). Bacteria obtained from 5 dph surviving larvae of all antibiotic groups (5-PM, 5-TC, 5-NB, and 5-EM) revealed that *Oceanobacter* (54.7–65.3%) was the dominant family, followed by *Marinobacter* (11.6–14.8%) (Fig 4A). Bacteria obtained from 20 and 40 dph surviving larvae in antibiotic groups showed that *Vibrio* (45.9–61.7%) was predominantly present in 20-TC and 40-TC, whereas *Alcanivorax* (37.1–61.8%) and *Marinobacter* (17.3–27.2%) were characteristically dominant in 20-NB and 40-NB and *Alcanivorax* (63.0–77.8%) was dominant in 20-EM and 40-EM (Fig 4B and 4C). Surviving larvae in 20-PM and 40-PM exhibited predominance of *Kordiimonas* (11.7–26.1%) and *Labrenzia* (11.6–13.2%), in contrast to the other three antibiotics (TC, NB, and EM).

### Biomarker ASVs associated with surviving and dead eel larvae

**Fig 5:**
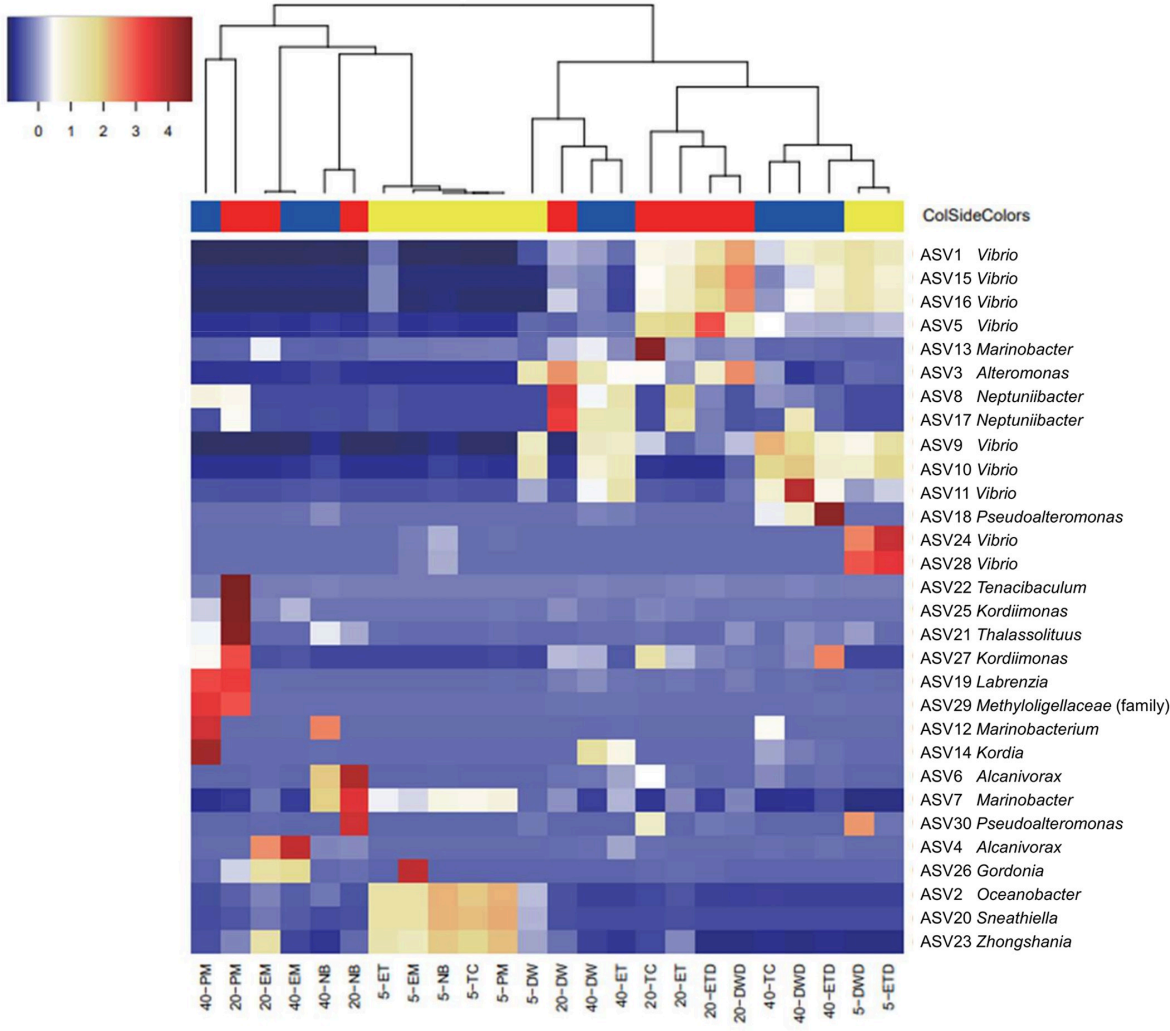
Heatmap of 30 most abundant ASVs detected in surviving and dead larvae at three growth stages. The colored columns below the dendrogram indicate larval samples at 5 dph in yellow, 20 dph in red and 40 dph in blue. Samples can be seen below the heatmap, ASVs and genus level taxonomy on the right side. Blue and red indicate low and high relative abundance of read numbers, respectively.

The distribution of the 30 most abundant ASVs detected in the surviving and dead larval samples is displayed as a heatmap for each experimental group at the genus level (Fig 5). Abundant ASVs of the surviving larvae in the antibiotic groups showed a trend that was drastically different from those of other groups. Specifically, dominant ASVs obtained from 5 dph larvae (5-PM, 5-TC, 5-NB, and 5-EM) differed from those obtained from 20 and 40 dph larvae. Among the antibiotic groups at 20 and 40 dph, the 20-PM and 40-PM, 20-NB and 40-NB, 20-EM and 40-EM groups clustered for each antibiotic. In contrast, the abundant ASVs of the surviving larvae in the 20-TC and 40-TC groups differed from those of the others and were similar to those of dead larvae in the control groups (DWD and ETD). Among the dead larvae in the control groups (DWD and ETD), ASVs belonging to *Vibrio* were detected in abundance in all their growth stages.

**Fig 6:**
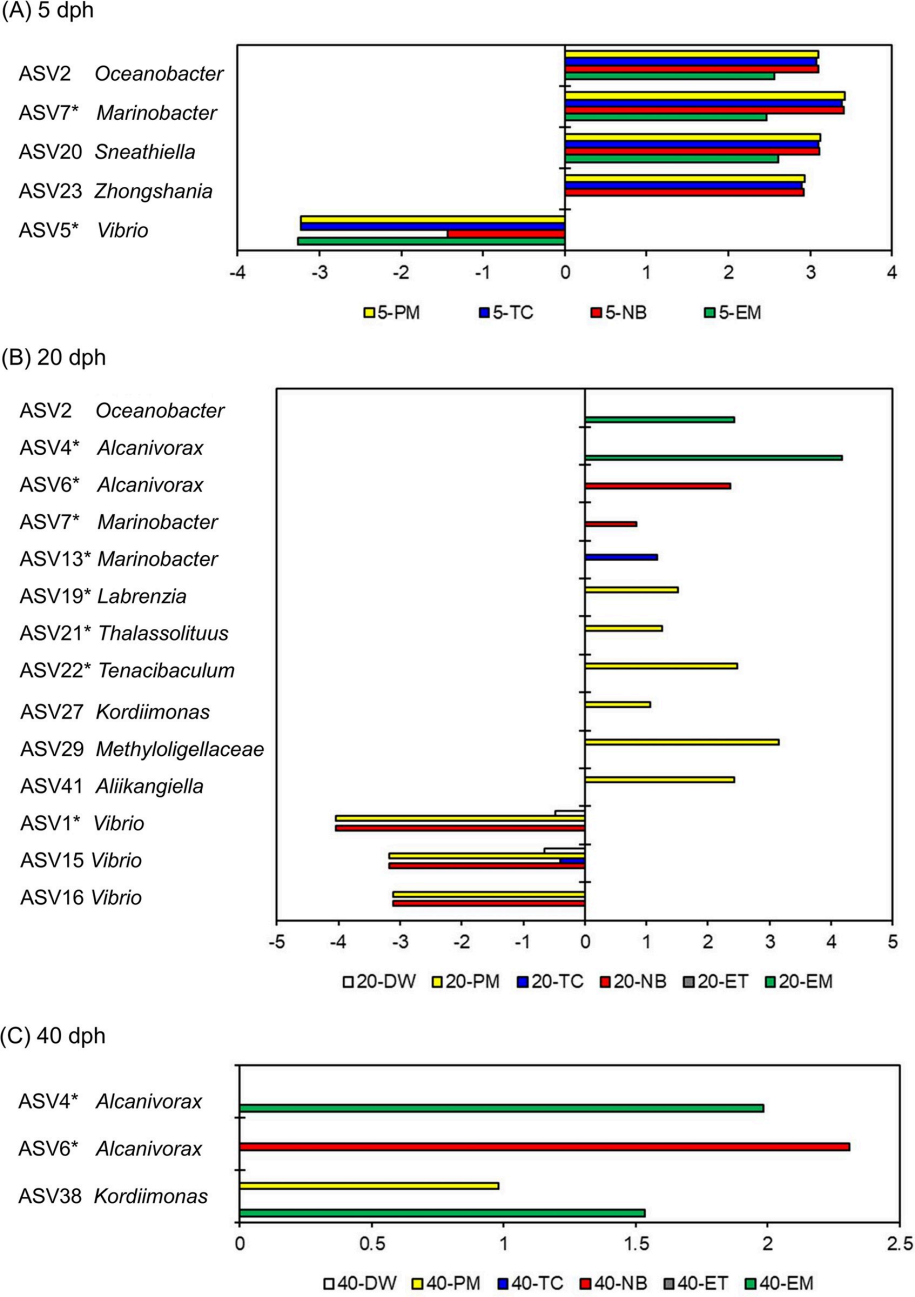
Extraction of biomarker ASVs enriched in surviving and dead larvae at three growth stage. (A) 5 dph, (B) 20 dph, and (C) 40 dph. For each ASV, surviving larvae in DW, PM, TC, NB were compared to dead larvae in DWD, whereas surviving larvae in ET and EM were compared to dead larvae in ETD. 5-DW and 5-ET were excluded from the statistical analysis, as only one sample was obtained (n = 1). The asterisks indicate biomarker ASVs isolated using a culture method.

ASVs with significantly different abundances between the surviving and dead larvae were detected at each growth stage (Fig 6). In all experimental groups, a total of 18 ASVs were identified as key biomarkers, including 5, 14, and 3 ASVs from 5, 20, and 40 dph, respectively. Fourteen ASVs with significantly increased abundance in the surviving larvae were classified into the following genera: *Oceanobacter* (ASV 2), *Alcanivorax* (ASV 4 and 6), *Marinobacter* (ASV 7 and 13), *Labrenzia* (now *Roseibium*; ASV 19), *Sneathiella* (ASV 20), *Thalassolituus* (ASV 21), *Tenacibaculum* (ASV 22), *Zhongshania* (ASV 23), *Kordiimonas* (ASV 27 and 38), *Methyloligellaceae* (family level; ASV 29), and *Aliikangiella* (ASV 41). All four ASVs (ASV 1, 5, 15, and 16) belonging to *Vibrio* exhibited significantly decreased abundance in the surviving larvae.

### Marine bacterial counts in eel larvae and the surrounding seawater

**Fig 7:**
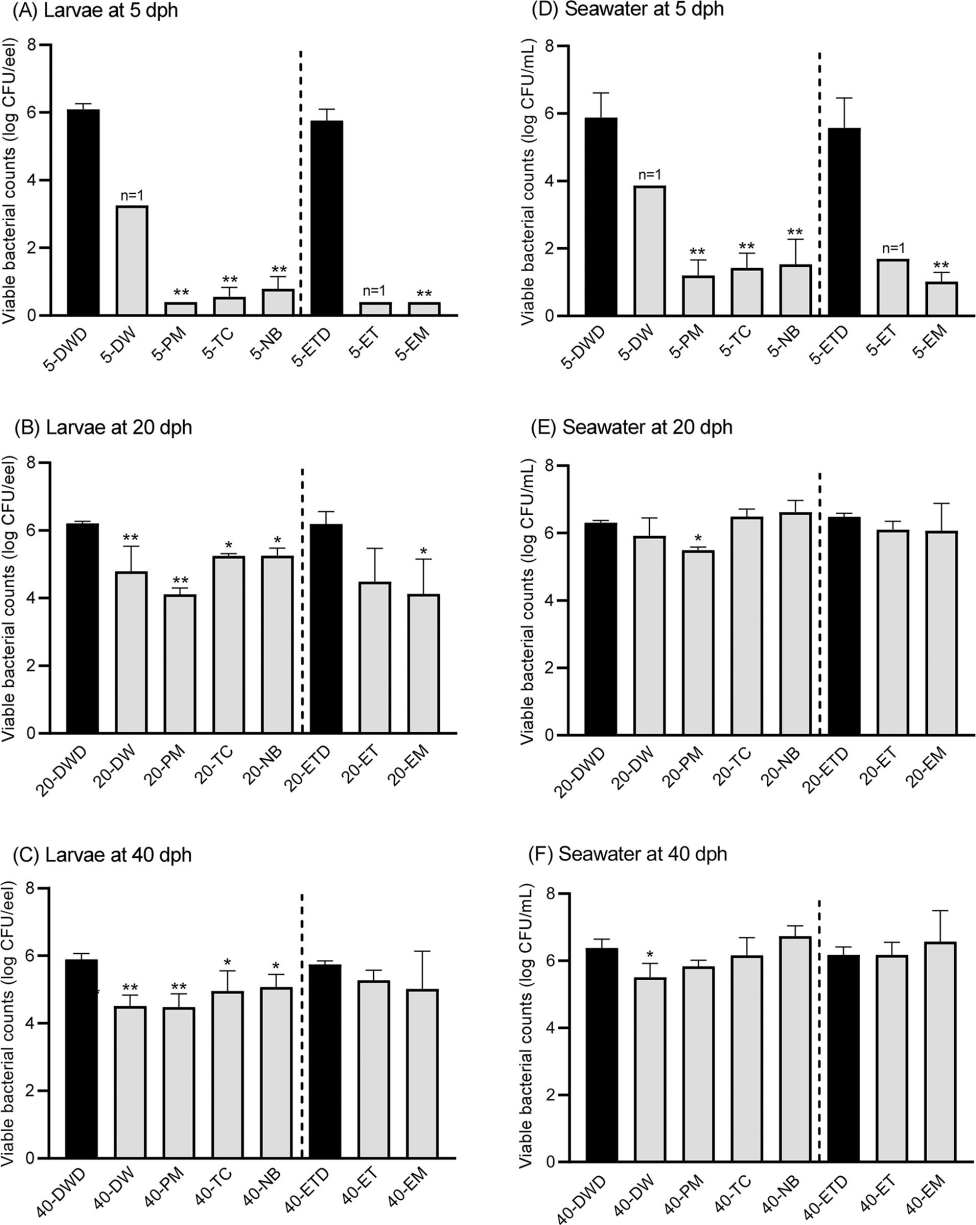
Marine bacterial counts in surviving and dead eel larvae and the surrounding seawater. The eel larval samples show at (A) 5 dph, (B) 20 dph, and (C) 40 dph. The seawater samples show at (D) 5 dph, (E) 20 dph, and (F) 40 dph. The bacterial counts of DW, PM, TC, and NB were compared to those of DWD, whereas the bacterial counts of ET and EM were compared to those of ETD. Data represent the mean ± standard deviation (n = 3), and the asterisks on the bars show significant differences (* *p* < 0.05; ** *p* < 0.01). 5-DW and 5-ET were excluded from the statistical analysis, as only one sample was obtained (n = 1).

Marine bacterial counts in surviving and dead larvae were compared for antibiotic and control groups (Fig 7A–7C). Through three growth stages, bacterial counts in dead larvae in control groups (DWD and ETD) were highest at 5.7–6.2 log CFU/eel. At 5 dph, bacterial counts in surviving larvae in all antibiotic groups (5-PM, 5-TC, 5-NB, and 5-EM) were < 0.8 log CFU/eel, which was significantly lower than those of dead larvae in the control groups (5-DWD and 5-ETD; *p* < 0.01) (Fig 7A). At 20 dph, the bacterial counts of surviving larvae in all antibiotic groups (20-PM, 20-TC, 20-NB, and 20-EM) ranged between 4.1–5.3 log CFU/eel, which was significantly different from those obtained from dead larvae in the control groups (20-DWD and 20-ETD; *p* < 0.05) (Fig 7B). At 40 dph, the bacterial counts of surviving larvae in the antibiotic groups (40-PM, 40-TC, and 40-NB), excluding 40-EM group, ranged from 4.5 to 5.1 log CFU/eel and were significantly lower than those in dead larvae in the control groups (40-DWD and 40-ETD; *p* < 0.05) (Fig 7C).

Marine bacterial counts in the surrounding seawater were compared between wells in which surviving larvae were observed in the antibiotic and control groups and wells in which dead larvae were observed in the control groups (Fig 7D–7F). During the three growth stages, bacterial counts from dead larvae in control groups (DWD and ETD) ranged from 5.6–6.5 log CFU/mL. At 5 dph, the bacterial counts in all antibiotic groups (5-PM, 5-TC, 5-NB, and 5-EM) were low at 1.0–1.5 log CFU/mL with a significant difference (*p* < 0.01) (Fig 7D). At 20 and 40 dph, no significant differences were observed in the antibiotic groups, except for 20-PM (Fig 7E and 7F).

### Isolation of bacterial strains from surviving and dead eel larvae

**Fig 8:**
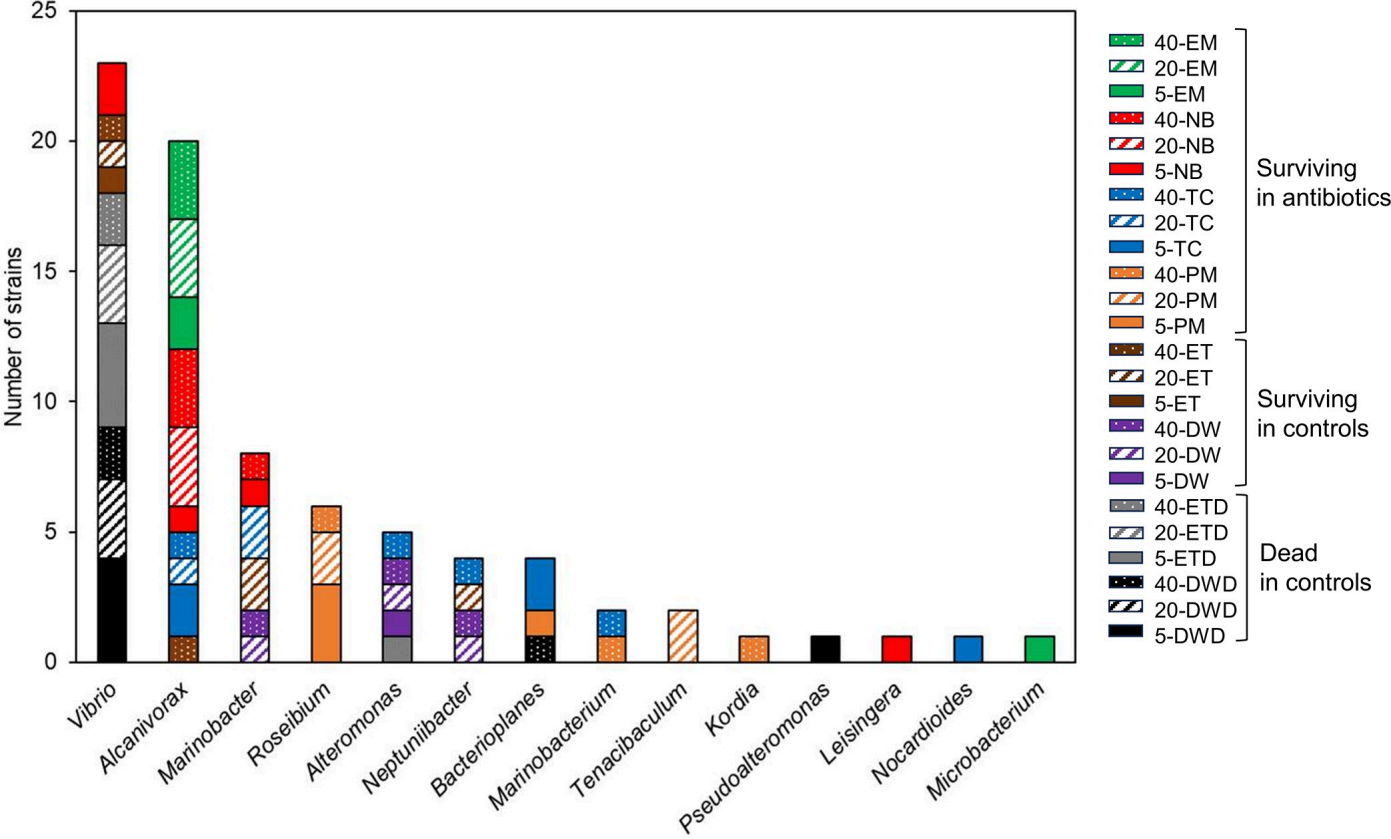
Numbers of bacterial isolates from eel larvae and the surrounding seawater assigned to genera level, based on partial 16S rRNA gene sequences. Surviving larvae in antibiotic groups (PM, TC, NB, and EM; n = 44), surviving larvae in control groups (DW and ET; n = 14), and dead larvae in control groups (DWD and ETD; n = 21).

Seventy-nine bacterial strains were predominantly isolated from eel larvae or seawater samples: 44 strains from surviving larvae or seawater in the antibiotic groups (PM, TC, NB, and EM), 14 strains from surviving larvae in the control groups (DW and ET), and 21 strains from dead larvae in the control groups (DWD and ETD) (Table 1 and Fig 8). Among the bacterial strains obtained from the surviving larvae in the antibiotic groups, the genus *Alcanivorax* (n = 19) were primarily isolated from TC, NB, and EM. The genus *Roseibium* (n = 6) was predominantly isolated from PM, whereas *Marinobacter* (n = 4) was isolated from TC and NB. Bacterial strains obtained from the surviving larvae in the control groups (DW and ET) primarily belonged to *Marinobacter* (n = 4), followed by *Alteromonas* (n = 3), *Neptuniibacter* (n = 3), and *Vibrio* (n = 3). Most of the bacteria isolated from dead larvae in the control groups (DWD and ETD) belonged to the genus *Vibrio* (n = 18).

**Table 1:** Identification of bacterial strains isolated from eel larvae and the surrounding seawater based on full-length 16S rRNA gene sequences.

Group	Representative	Accession No.	Closest type species	Accession No.	Similarity	No. of	Corresponding	Taxonomy assigned
	strains	(in this study)		(closest type species)	(%)	Strains	ASV ID	by Silva (QIIME2)
1	20DWE-22	LC797340	*Vibrio coralliilyticus*	NR_117892	99.5	12	1^b^	*Vibrio*
2	40TCE-21	LC797341	*Alteromonas abrolhosensis*	NR_181920	99.9	5	3	*Alteromonas*
			*Alteromonas macleodii*	CP003841	99.9			* *
3	40EME-11	LC797342	*Alcanivorax xiamenensis*	OP535888	99.9	10	4^b^	*Alcanivorax*
4	20ETE-22	LC797343	*Vibrio coralliilyticus*	NR_117892	99.9	1	5^b^	*Vibrio*
5	40TCE-11	LC797344	*Alcanivorax borkumensis*	MN186605	99.7	10	6^b^	*Alcanivorax*
6	5NBE-41	LC797345	*Marinobacter algicola*	NR_042807	99.8	6	7^b^	*Marinobacter*
7	40TCE-1	LC797346	*Neptuniibacter victor*	LC716006	98.3	4	8	*Neptuniibacter*
8	5DWE-12	LC797347	*Vibrio tubiashii*	CP009354	98.9	6	9	*Vibrio*
9	40DWE-24	LC797348	*Vibrio coralliilyticus*	NR_117892	99.4	1	11	*Vibrio*
10	40TCE-22	LC797349	*Marinobacterium jannaschii*	NR_113757	95.4	2	12	*Marinobacterium*
11	20TCE-31	LC797350	*Marinobacter adhaerens*	NR_074765	99.9	2	13^b^	*Marinobacter*
12	40PME-1	LC797351	*Kordia algicida*	NR_027568	99.8	1	14	*Kordia*
13	20PME-31	LC797352	*Roseibium aggregatum*	NR_113861	100.0	5	19^b^	*Labrenzia* ^c^
14	5TCE-12	LC797353	*Bacterioplanes sanyensis*	NR_126264	99.2	4	21^b^	*Thalassolituus*
15	20PME-11	LC797354	*Tenacibaculum aiptasiae*	NR_044202	99.9	2	22^b^	*Tenacibaculum*
16	5DWE-11	LC797355	*Vibrio tubiashii*	CP009354	99.6	2	24	*Vibrio*
17	5DWE-33	LC797356	*Pseudoalteromonas issachenkonii*	CP011030	100.0	1	30	*Pseudoalteromonas*
			*Pseudoalteromonas tetraodonis*	CP011041	100.0			* *
18	5DWE-32	LC797357	*Vibrio rotiferianus*	NR_118091	99.9	1	74	*Vibrio*
19	5PMS-11^a^	LC797358	*Roseibium album*	NR_042378	100.0	1	97	*Labrenzia* ^c^
20	5NBS-31^a^	LC797359	*Leisingera caerulea*	NR_118542	100.0	1	243	*Leisingera*
21	5TCS-31^a^	LC797360	*Nocardioides marinus*	NR_043787	99.3	1	634	*Nocardioides*
22	5EMS-12^a^	LC797361	*Microbacterium tenebrionis*	MW680833	99.4	1	-	*-*

^a^ The strains were isolated from seawater samples.

^b^ The ASV IDs were identified as biomarkers.

^c^ The genus *Labrenzia* was transferred to the genus *Roseibium* [31].

When the sequences of all the strains were compared with the ASV sequences, 78 strains were found to belong to 21 ASVs with 100% similarity (Table 1). In addition, 52 strains corresponded to 9 of the 18 ASVs selected as biomarkers. Full-length 16S rRNA gene sequences of the representative isolates were determined, and the most closely related species are listed in Table 1. In the surviving larvae, seven ASVs (ASV 4, 6, 7, 13, 19, 21, and 22) were significantly more frequent, whereas two ASVs (ASV 1 and 5) were significantly less abundant. ASV 4 and 6 were most closely related to *Alcanivorax xiamenensis* and *A*. *borkumensis*, respectively. ASV 7 and 13 were the most closely related to *Marinobacter algicola* and *M*. *adhaerens*, respectively. ASV 19 was most closely related to *Roseibium aggregatum*, ASV 21 to *Bacterioplanes sanyensis*, and ASV 22 to *Tenacibaculum aiptasiae*. In contrast, two ASVs (ASV 1 and 5) were most closely related to *Vibrio coralliilyticus*, based on both 16S rRNA gene and *pyrH* gene sequences (S2 Table).

## Discussion

In the present study, the effects of bacterial communities on eel larval survival were examined in laboratory experiments using various antibiotics. We found that closed-rearing systems, which are characterized by close relationships between hosts and microbiota, showed differences in bacterial characteristics between the surviving and dead larvae. We also successfully isolated biomarker strains that influence larval survival, enabling a detailed identification of the bacteria at the species level.

The four antibiotics, PM, TC, NB, and EM, significantly increased survival rates of larvae at 5 dph compared to the control groups (Fig 1A). The low survival rates of 5 dph larvae in the control groups could be attributed to other factors, such as floating mortality and low density. Unuma et al. [32] reported that when eel eggs were stocked in microplates, floating mortality was observed just after hatching, owing to the surface tension of seawater. They found that mortality can be suppressed with PEG addition, but PEG was not used in the present study, potentially resulting in an underestimation of the survival rate. At 5 dph, the bacterial counts of both surviving larvae and the surrounding seawater in the antibiotic groups were significantly lower than those in the dead larvae of the control groups (Fig 7A and 7D), indicating that the antibiotics caused a steady reduction in the bacterial counts. However, the associations between bacterial counts and larval survival at 5 dph was not comprehensively investigated due to the small number of surviving larval samples in the control groups. At 20 and 40 dph, most antibiotic groups showed significantly higher survival rates than the control groups (Fig 1B and 1C). The higher survival rates can be attributed to the bacterial counts in the surviving larvae; the counts were significantly lower than those in the surrounding seawater (Fig 7B, 7C, 7E, and 7F). The bacterial community structure of larvae at 5 dph differed from those at 20 and 40 dph. This difference can most likely be explained by larval differences in feeding history or the differences underlying their acclimation periods to the supplying seawater. This result is similar to that observed in European eel larvae, in which different bacterial communities are associated with pre-feeding and feeding larvae [20].

The majority of the top 30 ASVs detected from eel larvae in the present study belonged to the orders *Vibrionales*, *Oceanospirillales*, and *Alteromonadales* (Fig 5). These orders are consistent with some of the more frequent orders detected from European eel larvae at 9–28 dph [20]. The surviving larvae in the antibiotic groups and the dead larvae in the control groups hosted distinct bacterial communities (Figs 3A and 4). Moreover, ASV richness was significantly lower in the dead larvae (Fig 2A), indicative of *Vibrio* being the primary driver of larval mortality. These results are congruent with the fact that most of the bacterial strains isolated from dead larvae are of the genus *Vibrio* (Table 1 and Fig 8). Vibriosis, which is caused by the family *Vibrionaceae*, is one of the most common bacterial diseases affecting marine fish, especially larvae, resulting in high mortality [33, 34]. Because bacteria from *Vibrio* exhibit high affinity for eel larvae and have a high transmission rate, they are, overall, the main drivers of larval mortality. In contrast, *Vibrio* sequences were barely detected in antibiotic groups treated with PM, NB, and EM (Fig 4), indicating that these antibiotics have bactericidal activity against *Vibrio*. Administration of PM and EM is most effective in increasing the survival of carangid *Caranx mate* larvae, resulting in the suppression of *Vibrio* [35]. The survival rates among scallop larvae administered EM increased, and the corresponding populations of *Vibrio* spp. hosted by these larvae decreased [36]. NB has been shown to be the most sensitive antibiotic against *Vibrio* strains isolated from black-lipped pearl oysters [37]. The larval survival rates at 20 and 40 dph were significantly higher in TC group despite the high proportion of *Vibrio* (Fig 4). These results show that TC is also bacteriostatic against *Vibrio*, as TC typically exhibits bacteriostatic activity [38]. Overall, we suggest that the significant increase in larval survival with the addition of antibiotics can be attributed to: i) the non-dominance or reduced activity of *Vibrio* and ii) the dominance of bacterial communities that appeared in place of *Vibrio*.

All the four biomarker ASVs (ASV 1, 5, 15, and 16) enriched in dead larvae at 5 and 20 dph belonged to the genus *Vibrio* (Fig 6A and 6B). Among these ASVs, bacterial strains corresponding to ASV 1 and 5 were found to be the most closely related to *V*. *coralliilyticus* (Table 1 and S2 Table), a well-known coral pathogen [39] that is pathogenic to rainbow trout, *Artemia* nauplii, and larvae of oyster and scallop [40–42]. However, the pathogenicity of *V*. *coralliilyticus* strains in eel larvae has not yet been reported. A recent study also reported that *Vibrio* ASVs of the Harveyi clade reduce the survival of European eel larvae between 15 and 22 dph [20]. Therefore, *Vibrio* is likely to be one of the most important determinants of the survival of eel larvae during their early growth stages. Conversely, these *Vibrio* biomarkers might multiply rapidly after the death of the larvae; therefore, the pathogenicity of *Vibrio* strains, which correspond to the biomarker ASVs to eel larvae, need to be evaluated in the future.

Among the antibiotic groups, 14 ASVs were enriched in the surviving larvae, and the taxa were affiliated with diverse genera (Fig 6). Of the uncultured biomarker ASVs, the genus *Oceanobacter* (ASV 2), a known hydrocarbonoclastic bacterium with high n-alkane degrading activity, was the most dominant in oil spill microcosm [43]. Furthermore, *Oceanobacter* species have the ability to accumulate poly-*β*-hydroxybutyrate (PHB) [44, 45]. The genus *Sneathiella* (ASV20) was detected as indicator from brood pouch tissue and embryos during male pregnancy of pipefish [46]. *Zhongshania* (ASV 23) is dominant in sediments and seawater polluted with crude oil and aromatic compounds, and potentially degrade alkane and aromatics, respectively [47, 48]. The bacteria in the genus *Kordiimonas* (ASV 27 and 38) are common within coral holobiont and are thought to be one of the bacteria with important functions [49]. *Aliikangiella* (ASV 41) had a differentially abundant ASV in large size oysters than in small size oysters, indicating that it appears to have roles in nutrient absorption and energy acquisition [50].

In addition, bacterial strains corresponding to the seven biomarker ASVs were isolated from the surviving larvae (Table 1). *A*. *xiamenensis* (ASV 4) and *A*. *borkumensis* (ASV 6) were often detected in EM and NB groups, respectively, at 20 and 40 dph. The two bacterial species degrade a wide array of n-alkanes as their sole carbon source; specifically, the degradation of these alkanes is facilitated by alkane hydroxylase genes present in the two bacteria [51, 52]. *M*. *algicola* (ASV 7) and *M*. *adhaerens* (ASV 13) were first isolated from the dinoflagellate and marine aggregates of surface seawater, respectively [53, 54]. These bacteria are closely associated with marine phytoplankton or diatoms via the production of the siderophore vibrioferrin or aggregate formation in diatoms [55, 56]. Larvae at 20 dph subjected to PM revealed that *R*. *aggregatum* (ASV 19) plays an important role in marine biogeochemical cycles, such as denitrification, production of dimethylsulfoniopropionate (DMSP), and oxidation of carbon monoxide in marine environments [57–59]. *B*. *sanyensis* (ASV 21) was first isolated from a pool of marine *Spirulina platensis* cultivation, which is capable of accumulating PHB [60]. *T*. *aiptasiae* (ASV 22) was first isolated from a diseased sea anemone [61]. Thus, biomarker genera and species enriched in surviving larvae in the antibiotic groups appeared to be associated with marine plants, degradation or production of characteristic chemicals, and geochemical cycling of the marine environment. However, how these bacterial strains affect the survival and function of host eel larvae remains unclear. Future studies must delve into more fine-scale aspects of these bacterial strains. In addition, as this study did not include normal rearing feeding or seawater changes under well plates, the effects of these rearing conditions on the bacterial communities of eel larvae need to be studied in the future.

In conclusion, different antibiotic treatments in the laboratory led to significant variations in bacterial characteristics associated with surviving and dead larvae. Bacterial species of *Vibrio* were the cause of larval mortality. However, other bacterial communities altered by the addition of antibiotics could either be harmless or beneficial to larval survival. The identification of biomarker bacterial species associated with the survival of eel larvae offers valuable insights into improving larval survival in rearing systems. In the future, we aim to investigate the specific bacterial strains that are significantly enriched in both surviving and dead larvae to elucidate their effects on the survival of Japanese eel larvae.

## Supporting information

S1 Table Information of eel each sample and 16S rRNA gene amplicon sequences. (PDF)

S2 Table Identification of *Vibrio* strains isolated from eel larvae based on *pyrH* gene sequences. (PDF)
