# Supplementary material for: Microbial influence on the larval survival of Japanese eel Anguilla japonica: Antibiotic-mediated alterations and biomarker isolation
Source: PLoS One. 2024 Jul 8;19(7):e0306634. doi: 10.1371/journal.pone.0306634 (PMC11230566; doi:10.1371/journal.pone.0306634)
Supplement: S1 Table — (PDF) [file pone.0306634.s001.pdf]

S1 Table. Information of eel each sample and 16S rRNA gene amplicon sequences.

Sample ID	Major group	Substance	Exp. Number	Larval status	Larval growth stage	Number of reads
5-1-DWD	Control	Distilled water	1	Dead	5 dph	23,698
5-2-DWD	Control	Distilled water	2	Dead	5 dph	26,166
5-3-DWD	Control	Distilled water	3	Dead	5 dph	24,300
5-3-DW[a]	Control	Distilled water	3	Surviving	5 dph	21,387
5-1-PM	Antibiotic	Polymyxin B	1	Surviving	5 dph	19,474
5-2-PM	Antibiotic	Polymyxin B	2	Surviving	5 dph	19,126
5-3-PM	Antibiotic	Polymyxin B	3	Surviving	5 dph	22,966
5-1-TC	Antibiotic	Tetracycline	1	Surviving	5 dph	21,190
5-2-TC	Antibiotic	Tetracycline	2	Surviving	5 dph	18,646
5-3-TC	Antibiotic	Tetracycline	3	Surviving	5 dph	18,340
5-1-NB	Antibiotic	Novobiocin	1	Surviving	5 dph	21,479
5-2-NB	Antibiotic	Novobiocin	2	Surviving	5 dph	17,312
5-3-NB	Antibiotic	Novobiocin	3	Surviving	5 dph	25,429
5-1-ETD	Control	Ethanol	1	Dead	5 dph	25,363
5-2-ETD	Control	Ethanol	2	Dead	5 dph	22,618
5-3-ETD	Control	Ethanol	3	Dead	5 dph	26,158
5-2-ET[a]	Control	Ethanol	2	Surviving	5 dph	18,644
5-1-EM	Antibiotic	Erythromycin	1	Surviving	5 dph	17,182
5-2-EM	Antibiotic	Erythromycin	2	Surviving	5 dph	20,838
5-3-EM	Antibiotic	Erythromycin	3	Surviving	5 dph	16,496
20-1-DWD	Control	Distilled water	1	Dead	20 dph	39,658
20-2-DWD	Control	Distilled water	2	Dead	20 dph	29,402
20-3-DWD	Control	Distilled water	3	Dead	20 dph	30,671
20-1-DW	Control	Distilled water	1	Surviving	20 dph	24,820
20-2-DW	Control	Distilled water	2	Surviving	20 dph	30,518
20-3-DW	Control	Distilled water	3	Surviving	20 dph	30,481
20-1-PM	Antibiotic	Polymyxin B	1	Surviving	20 dph	31,959
20-2-PM	Antibiotic	Polymyxin B	2	Surviving	20 dph	29,314
20-3-PM	Antibiotic	Polymyxin B	3	Surviving	20 dph	31,554
20-1-TC	Antibiotic	Tetracycline	1	Surviving	20 dph	31,580
20-2-TC	Antibiotic	Tetracycline	2	Surviving	20 dph	30,809
20-3-TC	Antibiotic	Tetracycline	3	Surviving	20 dph	31,247
20-1-NB	Antibiotic	Novobiocin	1	Surviving	20 dph	27,757
20-2-NB	Antibiotic	Novobiocin	2	Surviving	20 dph	26,791
20-3-NB	Antibiotic	Novobiocin	3	Surviving	20 dph	29,885
20-1-ETD	Control	Ethanol	1	Dead	20 dph	23,643
20-2-ETD	Control	Ethanol	2	Dead	20 dph	27,734
20-3-ETD	Control	Ethanol	3	Dead	20 dph	27,944
20-1-ET	Control	Ethanol	1	Surviving	20 dph	22,421
20-2-ET	Control	Ethanol	2	Surviving	20 dph	27,028
20-3-ET	Control	Ethanol	3	Surviving	20 dph	26,466
20-1-EM	Antibiotic	Erythromycin	1	Surviving	20 dph	24,045
20-2-EM	Antibiotic	Erythromycin	2	Surviving	20 dph	26,076
20-3-EM	Antibiotic	Erythromycin	3	Surviving	20 dph	20,557
40-1-DWD	Control	Distilled water	1	Dead	40 dph	31,416
40-2-DWD	Control	Distilled water	2	Dead	40 dph	30,755
40-3-DWD	Control	Distilled water	3	Dead	40 dph	34,648
40-1-DW	Control	Distilled water	1	Surviving	40 dph	32,672
40-2-DW	Control	Distilled water	2	Surviving	40 dph	28,109
40-3-DW	Control	Distilled water	3	Surviving	40 dph	29,692
40-1-PM	Antibiotic	Polymyxin B	1	Surviving	40 dph	28,534
40-2-PM	Antibiotic	Polymyxin B	2	Surviving	40 dph	28,006
40-3-PM	Antibiotic	Polymyxin B	3	Surviving	40 dph	28,947
40-1-TC	Antibiotic	Tetracycline	1	Surviving	40 dph	28,142
40-2-TC	Antibiotic	Tetracycline	2	Surviving	40 dph	29,032
40-3-TC	Antibiotic	Tetracycline	3	Surviving	40 dph	28,316
40-1-NB	Antibiotic	Novobiocin	1	Surviving	40 dph	21,859
40-2-NB	Antibiotic	Novobiocin	2	Surviving	40 dph	29,677
40-3-NB	Antibiotic	Novobiocin	3	Surviving	40 dph	25,902
40-1-ETD	Control	Ethanol	1	Dead	40 dph	28,732
40-2-ETD	Control	Ethanol	2	Dead	40 dph	24,307
40-3-ETD	Control	Ethanol	3	Dead	40 dph	30,072
40-1-ET	Control	Ethanol	1	Surviving	40 dph	28,552
40-2-ET	Control	Ethanol	2	Surviving	40 dph	34,462
40-3-ET	Control	Ethanol	3	Surviving	40 dph	36,669
40-1-EM	Antibiotic	Erythromycin	1	Surviving	40 dph	31,918
40-2-EM	Antibiotic	Erythromycin	2	Surviving	40 dph	29,570
40-3-EM	Antibiotic	Erythromycin	3	Surviving	40 dph	23,515

[a] The sample was obtained from one experiment ($n = 1$).
