# Supplementary material for: Microbial influence on the larval survival of Japanese eel Anguilla japonica: Antibiotic-mediated alterations and biomarker isolation
Source: PLoS One. 2024 Jul 8;19(7):e0306634. doi: 10.1371/journal.pone.0306634 (PMC11230566; doi:10.1371/journal.pone.0306634)
Supplement: S2 Table — (PDF) [file pone.0306634.s002.pdf]

S2 Table. Identification of *Vibrio* strains isolated from eel larvae based on *pyrH* gene sequences.

Group	Representative strains	Accession No. (in this study)	Closest type species	Accession No. (closest type species)	Similarity (%)	Corresponding ASV ID
1	20DWE-22	LC797362	*Vibrio coralliilyticus*	GU266292	98.7	1[a]
4	20ETE-22	LC797363	*Vibrio coralliilyticus*	GU266292	99.8	5[a]
8	5DWE-12	LC797364	*Vibrio neptunius*	CP079859	96.3	9
9	40DWE-24	LC797365	*Vibrio coralliilyticus*	GU266292	98.7	11
16	5DWE-11	LC797366	*Vibrio neptunius*	CP079859	98.1	24
18	5DWE-32	LC797367	*Vibrio campbellii*	LR860934	99.8	74

[a] The ASV IDs were identified as biomarkers.
